# The role of a radiopaque peri-rectal hydrogel spacer in aiding accurate daily image-guidance for prostate stereotactic radiotherapy

**DOI:** 10.3389/fonc.2024.1386058

**Published:** 2024-06-18

**Authors:** Oded Icht, Shir Schlosser, Miriam Weinstock-Sabbah, Mor Rephael, Dimitri Bragilovski, Assaf Moore, Tzippora Shochat, Dror Limon, Elisha Fredman

**Affiliations:** ^1^ Department of Radiation Oncology, Davidoff Cancer Center, Rabin Medical Center, Petah Tikvah, Israel; ^2^ School of Medicine, Tel-Aviv University, Tel-Aviv, Israel; ^3^ Department of Biostatistics, Rabin Medical Center, Petah Tikvah, Israel

**Keywords:** prostate, radiation, fiducial markers, hydrogel, SpaceOAR

## Abstract

**Introduction:**

Precise patient positioning with image guidance (IGRT) is essential for safe prostate radiotherapy. We present the first report of utilizing a CT-visible hydrogel spacer, used to decrease rectal radiation dose, as a surrogate fiducial marker to aid in daily IGRT with cone-beam CT (CBCT) in stereotactic radiotherapy (SABR) for prostate cancer.

**Materials and methods:**

Prior to CT simulation, patients underwent placement of three intraprostatic gold fiducial markers and radiopaque hydrogel spacer per standard practice. At treatment, after initial setup, a CBCT was acquired and fused to the planning CT based on 3-dimensional matching of the spacer. A second alignment was then performed based on the fiducial markers. The six directional shifts (three linear and three rotational) were recorded, and the differences compared.

**Results:**

140 individual fractions across 41 consecutive patients were evaluated. Mean/median differences between hydrogel spacer-based and fiducial-based alignment in linear (vertical, longitudinal, lateral) and rotational (rotation, pitch, roll) shifts were 0.9/0.6mm, 0.8/0.5mm, and 0.6/0.4mm, and 0.38/0, 0.62/0, and 0.35/0 degrees, respectively. No difference was observed in 9.9%, 22.9%, and 22.14% of linear shifts, and 65.7%, 65%, and 66.4% rotational shifts, respectively. Significantly smaller differences were observed in the latter 70 fractions vs. the former, and results were consistent across evaluators.

**Conclusions:**

For precise daily IGRT with CBCT for prostate SABR, alignment using a radiopaque hydrogel spacer was highly comparable to intraprostatic fiducial markers. This represents the first report supporting an additional indication of IGRT for a CT-visible hydrogel spacer, to further enhance treatment accuracy and potentially obviate the need for the additional fiducial marker procedure.

## Introduction

Delivering high dose radiation therapy for the treatment of localized adenocarcinoma of the prostate has been demonstrated in multiple prospective clinical trials to improve biochemical progression free survival (bPFS) relative to historical regimen (1–4). Dose-escalation, however, can potentially increase the risks of treatment-related side effects due to the close proximity of normal structures such as the bladder and rectum (1–3).

One method to limit high-dose radiation exposure to the rectum is transperineal insertion of a polyethylene glycol hydrogel into the potential fat space between Denonvilliers’ fascia and the anterior rectal wall. Hydrogel placement prior to prostate radiotherapy has been demonstrated to be an effective method of decreasing the risk of high-grade rectal toxicity (5–9) with very low rates of reported complications (10). A newer iteration of one form of hydrogel spacer, contains a component of bound iodine making it easily visible on CT clearly demarcating the posterior prostate and anterior rectal wall (11).

An essential aspect of accurate dose delivery, in particular for stereotactic radiotherapy (SABR), is the utilization of highly conformal radiation fields (12, 13) paired with daily image guidance (IGRT) to ensure proper targeting of the regions at-risk and avoidance of the surrounding structures (14). Aligning to the prostate gland, which is situated among comparatively isodense tissues, can potentially be imprecise with inter-observer variability (15). To enhance the reproducibility of IGRT, gold fiducial markers can be implanted into the prostate, serving as readily identifiable reference points within the target volume (15–17). Fiducial marker placement, however, entails procedural time, often anesthesia time, and can result in urinary complications depending on the method used, such as bleeding (18), or infection (19, 20), with reported rates of high-grade complications as high as 3% in the context of trans-rectal insertion.

Currently, fiducial markers and hydrogel spacers each serve their own distinct purposes. As a radiopaque 3-dimensioal stable structure immediately posterior to the prostate and demarcating the anterior rectal wall, the hydrogel spacer has the potential to additionally serve as a surrogate or supporting marker in daily IGRT. One injectable material serving a dual purpose could potentially decrease procedure time, discomfort, risk, and cost. To date, the accuracy and reproducibility of hydrogel spacer-based IGRT in prostate SABR has not been objectively reported in the literature. To this end, we prospectively tested the hypothesis that target alignment during modern dose-escalated prostate stereotactic radiotherapy using cone-beam CT (CBCT) could be performed using the reference of a hydrogel spacer with accuracy comparable to the gold standard of intra-prostatic fiducial markers, as defined by linear and rotational shift differences of ≤1mm and ≤1 degree, respectively.

## Materials and methods

Internal review board approval was obtained for analysis of prospectively collected data regarding IGRT for men undergoing SABR in the treatment of prostate cancer. As per standard departmental practice, patients without contraindication underwent placement of three gold fiducial markers (Civco Radiotherapy, Orange City, IA, USA) and a hydrogel spacer (Boston Scientific, Marlborough, MA, USA) (standard volume 10cc) using local anesthesia in the department of radiation oncology. Both were inserted transperineally under direct transrectal ultrasound (TRUS) guidance and visualization to confirm appropriate deployment of the fiducial markers bilaterally in the prostate and a minimum of 10mm posterior displacement of the rectum at center-midgland by the hydrogel.

The following week (between 6–8 days after), patients completed CT (Toshiba, Model: Aquillion RT(and MRI simulation (Siemens, VIDA) using standard immobilization (indexed knee and ankle supports, arms across chest), and treatment planning to deliver 5-fraction SABR using volumetric arc therapy (VMAT) was performed. Bowel preparation before simulation and each treatment comprised a daily laxative and a glycerol-based enema within 1–2 hours. The clinical target volume (CTV) was defined as the prostate gland and the seminal vesicles (SV; for intermediate and high-risk cases) with an isotropic 5mm planning target volume (PTV) expansion, except posteriorly where it was 3mm. To account for SV motion/variability, position on both planning CT and MRI was taken into consideration resulting in a planning ITV for this structure. For high and very-high risk cases, the CTV included the pelvic lymph nodes (common, internal and external iliac, presacral and obturator), contoured as a 7 mm volume around the corresponding blood vessels, while excluding adjacent organs (muscle, bone, bowel), with an additional PTV expansion of 5 mm. The proximal 1–2cm of SV in favorable intermediate risk cases and the entire SV in unfavorable intermediate risk were included. Presence of extracapsular extension was only considered a contraindication to hydrogel placement if located in the posterior or posterolateral aspects of the prostate gland. SABR fractions were scheduled every-other-day.

At treatment, usual patient setup was first performed based on skin tattoos and pre-recorded shifts, followed by acquisition of a CBCT (Varian, Model: TrueBeam, CBCT Scan Pelvis protocol. Software: Varian, TPS: Eclipse, Ver 16.1) in LINAC-based prostate SABR. Two sets of alignments were done, first matching the spacer on CBCT to that on the treatment planning CT in 3-dimensions with 6-degrees of directional freedom, utilizing both the LINAC auto alignment function and manual adjustment as needed. The exact positioning shifts from initial setup (three linear in mm, three rotational in degrees), were recorded. A second alignment was then performed to the fiducial markers on CBCT with those on the planning CT without regard for the spacer, and the values of those adjustments were similarly recorded. Spacer-based and fiducial-based auto- and manual-alignment techniques without confounding data from the second material was achieved by adjusting the clip-box fusion field to encompass exclusively the radiopaque hydrogel followed by the fiducial markers, respectively. The process was performed sequentially by the treating therapist followed by the radiation oncologist who performed the fiducial marker/hydrogel insertion for inter-observer comparison, and later repeated offline by the treating physician for assessment of intra-observer reliability.

The differences in linear and rotational shifts between the two methodologies were reported using descriptive statistics and analyzed with a paired t-test for equivalence with a TOST level of 0.05 for equivalence using pre-specified criteria of 1mm and 1 rotational degree. Variations between means from the first to the second half of the study period were assessed with 2-way ANOVA, and a regression analysis was performed to evaluate the influence of different shift types and prostate volume. Intra- and inter-observer reliability of assessors was evaluated using standard error of measurement (SEM) and intraclass correlation coefficient (ICC). Potential dosimetric changes resulting from differences between alignment methods were assessed.

## Results

**Figure 1:**
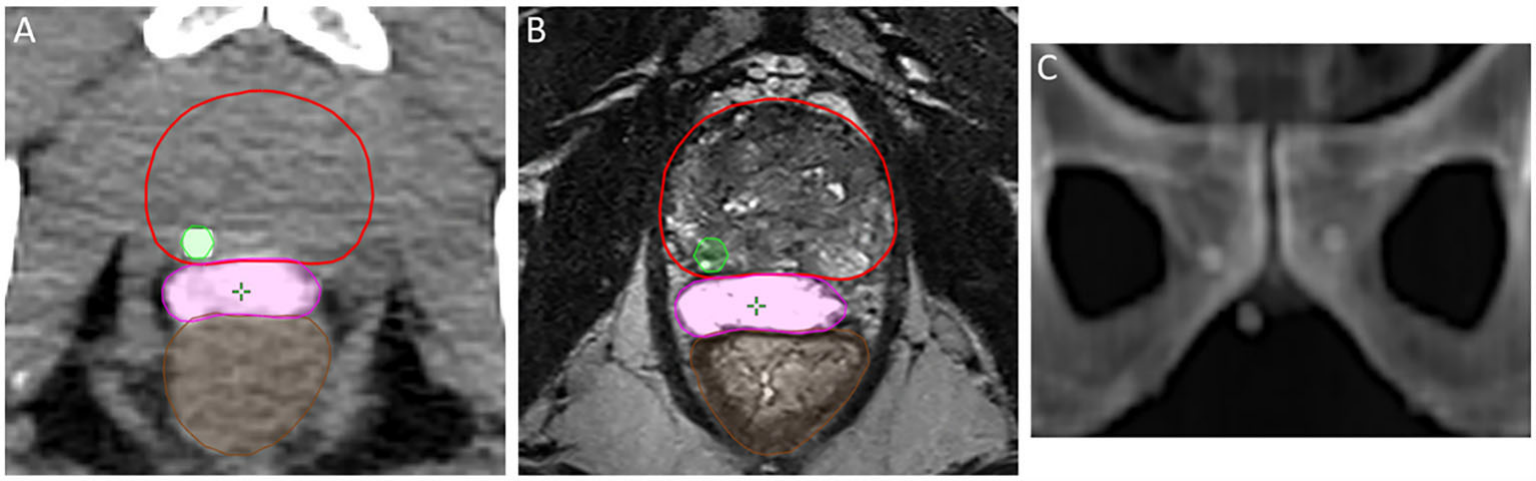
Proper placement of the hydrogel spacer (magenta) achieving posterior rectal displacement, and location of gold fiducial marker in the prostate parenchyma (green) on axial non-contrast CT **(A)** and axial T2-weighted MRI **(B)**. Multi-planar spacing of fiducial markers as seen on DRR **(C)**.

**Figure 2:**
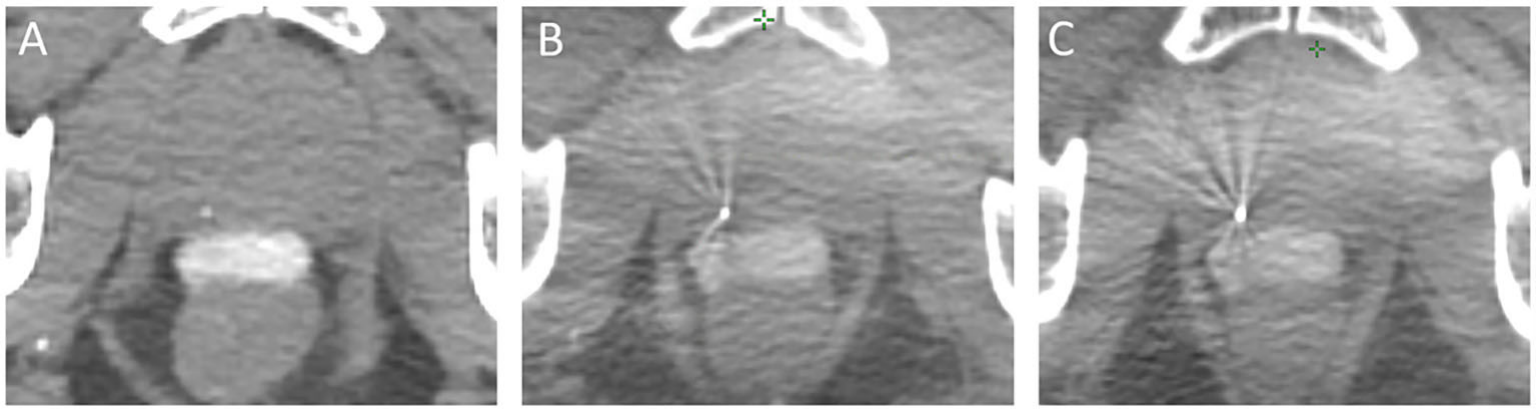
Stability of the hydrogel spacer material from simulation **(A)** through first **(B)** and last **(C)** fraction CBCT.

Over a period of 16 weeks, 140 individual fractions from 41 consecutive patients were recorded and analyzed (n=140). The number of included fractions out of total delivered (n=205) was only limited by availability of the study physicians to be present at the exact time of treatment setup and no fractions were intentionally excluded. Patient and tumor characteristics are presented in **Table 1**. In all instances the procedure of fiducial marker and hydrogel spacer placement was successful without complication (**Figure 1**). The majority of fractions were given for intermediate-risk disease (68.3%), followed by high-risk (21.9%), and low-risk (9.7%). While not directly measured, hydrogel shape and volume remained consistent throughout treatment as assessed qualitatively (**Figure 2**).

**Table 1:** Patient characteristics.

Variable	n (%)
**All patients**	41 (100)
**Age, years (range)**	73 (50 - 81)
T stage*	
1c	38 (92.6)
2c	1 (2.4)
3a	2 (4.8)
PSA (ng/mL)	
<10	28 (68.3)
10–20	9 (21.9)
>20	4 (9.7)
Grade group*	
1	4 (9.7)
2–3	32 (78)
4–5	5 (12.2)
Risk Group*	
Low	4 (9.7)
Intermediate	28 (68.3)
High	9 (21.9)
Prostate volume (cc)	
15–30	10 (24.3)
31–60	24 (58.5)
> 60	7 (17)
Target volume	
Prostate	3 (7.3)
+pSV	15 (36.5)
+SV	15 (36.5)
+SV/+LN	8 (19.5)
Systemic Therapy	
None	18 (43.9)
ADT x 6 months	15 (36.5)
ADT x 18 months	8 (19.5)

*Per National Comprehensive Cancer Network (NCCN) guidelines.

PSA, prostate specific antigen; pSV, proximal seminal vesicle; LN-lymph nodes; ADT, androgen deprivation therapy.

The mean and median delta between spacer-based and fiducial-based alignment for vertical, longitudinal, and lateral shifts were 0.9/0.6mm (90% CL mean -0.0294 – 0.00979, p < 0.001), 0.8/0.5mm (90% CL mean -0.00260 – 0.0312, p < 0.001), and 0.6/0.4mm (90% CL mean -0.00657 – 0.0380, p < 0.001), respectively, and for rotation, pitch, and roll shifts were 0.38/0 (90% CL mean -0.1183 – 0.2197, p < 0.001), 0.62/0 (90% CL mean -0.0391 – 0.5049, p < 0.001), and 0.35/0 (90% CL mean -0.0110 – 0.2339, p < 0.001) degrees, respectively (**Table 2**). Numerically no difference was observed between methods in 9.9%, 22.9%, and 22.14% of linear shifts, and 65.7%, 65%, and 66.4% rotational shifts, respectively. Prostate volume did not significantly correlate with shift differences in any of the six axes (correlation coefficient range -0.06 – 0.21), nor did spacer height or treatment field. Good intra-observer (within the treating physician’s live and offline alignments) and inter-observer (between therapist and physician) reliability was observed, with ICC and SEM values of 0.98 and 0.04.

**Table 2:** Directional shifts characterizing the quantitative differences between hydrogel spacer-based vs. fiducial-based daily target alignment.

Axis	SpaceOAR shift (Mean ± std)	Fiducials shifts (Mean ± std)	Delta shifts (Mean ± Std)	90% CL Mean	SpaceOAR shift (IQR)	Fiducials shifts (IQR)	Median shifts (IQR)	P value
Linear (mm)								
**Vertical**	4.1 ± 3.3	4.2 ± 3.3	0.9 ± 0.9	-0.0294 – 0.00979	3.15 (1.6–5.6)	3.2 (1.6–6.3)	0.6 (0.2–1.2)	<0.001*
**Longitudinal**	4.1 ± 3.9	3.95 ± 3.04	0.8 ± 0.9	-0.00260 – 0.0312	3.25 (1.8–5.4)	3.2 (1.8–5.4)	0.5 (0.1–1.3)	<0.001*
**Lateral**	5.27 ± 4.3	5.05 ± 4.31	0.6 ± 1	-0.00657 – 0.0380	4.75 (1.9–7.6)	4.45 (1.8–7.2)	0.4 (0.1–0.8)	<0.001*
Rotational (deg.)								
**Rotation**	1.3 ± 1.8	1.2 ± 1.9	0.38 ± 1.15	-0.1183 – 0.2197	0.8 (0.4–1.3)	0.8 (0.4–1.4)	0 (0–0.3)	<0.001*
**Pitch**	2.5 ± 3.2	2.2 ± 2.9	0.62 ± 1.85	-0.0391 – 0.5049	1.3 (0.6–2.9)	1.3 (0.6–2.8)	0 (0–0.45)	<0.001*
**Roll**	1.2 ± 1.1	1 ± 0.8	0.35 ± 0.81	-0.0110 – 0.2339	0.9 (0.9–1.5)	0.9 (1.3–1.5)	0 (0–0.4)	<0.001*

Std, standard deviation; IQR, interquartile range.

A favorable learning curve was observed as experience with the novel alignment method was gained, with mean differences between alignment methods significantly smaller in the second half of the study period compared to the first across five of the six shift directions (**Table 3**). A finding of no difference was also notably more frequent among the second half of fractions compared to the first (58.6% vs 25.2%), in particular for rotation (92.7%), pitch (94.3%), and roll (92.9%).

**Table 3:** Degree of directional shift adjustments between first vs. second half of study period demonstrating learning curve in hydrogel spacer-based alignment technique.

Axis	Mean: Fx 1–70	Mean: Fx 71–140	P value
Linear (mm)			
**Vertical**	1.1	0.6	0.0032*
**Longitudinal**	1.0	0.7	0.0464*
**Lateral**	0.8	0.5	0.1244
Rotational (deg.)			
**Rotation**	0.7	0.02	<0.0001*
**Pitch**	1.2	0.8	<0.0001*
**Roll**	0.65	0.05	<0.0001*

Fx, fraction.

Among the six shift directions, pitch was found to be the most variable, with 25^th^ and 75^th^ interquartile ranges of 0 and 0.45 degrees. These differences were observed to correlate closely with daily rectal emptying. When rotational shifts were minimized, however, linear shifts were also optimized, as seen by mean differences of 0.6 ± 0.7 mm, 0.8 mm, and 0.4 mm for vertical, longitudinal, and lateral shifts in fractions where no rotational delta was observed.

## Discussion

Herein we present the first objective report, to the best of our knowledge, demonstrating the ability of a radiopaque hydrogel spacer, known to significantly reduce rectal dose and consequently rectal toxicity (5–9), to simultaneously serve an additional role in daily IGRT using CBCT for prostate SABR. Alignment to the hydrogel spacer in this prospectively collected analysis revealed a high degree of fidelity to the gold standard of implanted fiducial markers. As evidence of its increasing pertinence, the first prospective trial attempting to study this potential application was recently registered (clinicaltrials.gov ID NCT05650021, not yet accruing as of submission).

A number of important findings emerged in our cohort of 140 fractions. First, the mean and median deltas in all six shift directions across the study period were minimal, with sub-millimeter linear shift and sub-degree rotational shift differences, meeting pre-specified criteria. Within the context of standard PTV expansions applied in contemporary trials of prostate SABR which typically range from 3–5mm (21, 22) the observed differences between the techniques would not be expected to be of clinical significance, as supported by the findings of statistically equivalent dosimetry. Second, a learning curve of this IGRT method emerged over the study period, demonstrating that without specific training, combining or modifying IGRT techniques by incorporating alignment to the hydrogel spacer is readily feasible with improving accuracy. Thirdly, the importance of proper bowel preparation was highlighted in our experience as an influential contributing factor to changes in pitch, no matter the IGRT technique.

Minimizing the number and length of procedures is an important component of improving the safety and efficiency of quality patient-centered care. Implanting a material that serves multiple purposes can decrease procedure time, degree and extent of anesthesia, and cost. Unlike the hydrogel which is injected in the fat-space posterior to the prostate, fiducial placement necessitates multiple violations of the prostatic capsule. Loh et al. found a patient-reported rate of dysuria and urinary frequency of 27% after fiducial placement (19), and Mendenhall et al. reported a 1.5% rate of bacterial infection necessitating hospitalization after a trans-rectal fiducials insertion, representing a non-insignificant risk of a high-grade complication (20), with increased rates over time, theorized to result from growing antibiotic resistance (23, 24). Even with the more modern transperineal approach for fiducial placement, Jorogo et al. reported a 14% risk of post-procedural hematuria (18), supporting the continued non-insignificant risks associated with needle introduction into the prostate. Data support recent prostate biopsy as an additional risk factor for infection (25) after fiducial marker placement, relevant to practically all patients. In contrast, the most recent report from the MAUDE (Manufacturer and User Facility Device Experience database) demonstrates a rate of complications (misplacement, abscesses, fistulas) following and directly related to hydrogel spacer insertion of <0.01% (10). Specifically regarding hyaluronic acid spacers, there may also be the potential to dissolve the implanted material with hyaluronidase, thereby reducing the risk of serious complications (26). As such, the potential to utilize a hydrogel spacer as an IGRT tool may help mitigate risk of complications such as hematuria or infection, in addition to improving efficiency and throughput. While to date the exact risk of post-operative infection after hydrogel placement is unknown, it is reasonable to posit that omitting one of two invasive procedures can help decrease the overall risk.

In addition to serving as an IGRT aid on CBCT published experiences have demonstrated the reliable consistency of the shape, volume, and density of hydrogel spacer, in particular the SpaceOAR™, over the length of both brief and protracted radiotherapy (27, 28), a finding qualitatively appreciated in our series (**Figure 2**). Furthermore, while the clinical implications are unknown, the existence of multiple solid markers within the prostate gland has been shown to cause calculable dose perturbations, dependent on marker size, material, orientation, and incident beam energy (29). At this time, such perturbations have not been demonstrated quantitatively with hydrogel spacers, though this phenomenon in comparison to metallic markers has not been rigorously studied.

Importantly, the ability of the spacer to aid in IGRT would be expected to be broadly applicable to most clinical scenarios. There was no correlation between setup consistency and prostate volume, and accuracy of the novel methodology was seen in SABR targeting the prostate alone, SVs, and pelvic lymph nodes. Newer technologies have been reported to enhance intra-fraction accuracy using either live- or interval timed-tracking of fiducial markers (30), or of the prostate itself (31). These can be further methods for enhancing precision during delivery of radiation therapy, though some of these abilities are not widely available in many centers globally. As such, IGRT as studied here can serve an important role for such cohorts.

This first-in-literature analysis of hydrogel-based IGRT on CBCT for prostate SABR has multiple strengths, including being a robust prospective cohort performed and collected at a large academic medical center and over a relatively short period of time. Treatment planning in all cases incorporated MRI simulation to enhance target definition and accuracy, and treatment was prescribed to contemporary SABR dosing with modern VMAT techniques. Target alignment was based both on automatic LINAC-based fusion between planning CT and CBCT and manual adjustments made in real time by the treating team of therapists and physicians. Limitations of the study include being performed at a single institution, individual variation in the success of bowel preparation prior to each fraction, impact of imaging quality based on body habitus, as well as a degree of inherent uncertainty within image matching as a result of the image quality and windowing differences between planning CT and CBCT imaging. Additionally, the difference in actual set-up time impacted by each method of IGRT was not quantitatively compared so conclusions cannot be drawn as to whether the hydrogel spacer method may increase overall length of treatment.

## Conclusions

In conclusion, IGRT using CBCT for prostate SABR with reference to a radiopaque hydrogel spacer was highly comparable to, and consistent with, the gold standard of implanted intraprostatic fiducial markers, with an observed rapid learning curve among operators. This represents the first report supporting an additional indication of IGRT for a CT-visible hydrogel spacer, to further enhance treatment accuracy and potentially obviate the need for the additional fiducial marker procedure.

## Data availability statement

The raw data supporting the conclusions of this article will be made available by the authors, without undue reservation.

## Ethics statement

The studies involving humans were approved by Internal review board - Rabin Medical Center. The studies were conducted in accordance with the local legislation and institutional requirements. The ethics committee/institutional review board waived the requirement of written informed consent for participation from the participants or the participants’ legal guardians/next of kin because this study was not prospective in nature and all treatments were delivered per current standards of care.

## Author contributions

OI: Conceptualization, Data curation, Formal analysis, Writing – original draft, Writing – review & editing. SS: Data curation, Writing – review & editing. MW: Data curation, Investigation, Writing – review & editing. MR: Investigation, Writing – review & editing. DB: Investigation, Resources, Supervision, Writing – review & editing. AM: Data curation, Formal analysis, Investigation, Writing – review & editing. TS: Formal analysis, Methodology, Writing – review & editing. DL: Supervision, Writing – review & editing. EF: Conceptualization, Formal analysis, Investigation, Methodology, Resources, Supervision, Writing – original draft, Writing – review & editing.
